# Editorial: Safety, Efficacy and Mechanisms of Action of Mesenchymal Stem Cell Therapies

**DOI:** 10.3389/fimmu.2020.00243

**Published:** 2020-02-18

**Authors:** Guido Moll, Martin J. Hoogduijn, James A. Ankrum

**Affiliations:** ^1^BIH Center for Regenerative Therapies (BCRT), Charité Universitätsmedizin Berlin, Corporate Member of Freie Universität Berlin, Humboldt-Universität zu Berlin, Berlin Institute of Health (BIH), Berlin, Germany; ^2^Nephrology and Transplantation, Department of Internal Medicine, Erasmus MC, University Medical Center, Rotterdam, Netherlands; ^3^Roy J. Carver Department of Biomedical Engineering, Fraternal Order of Eagles Diabetes Research Center, Pappajohn Biomedical Institute, University of Iowa, Iowa City, IA, United States

**Keywords:** mesenchymal stem cells, mesenchymal stromal cells, immunomodulation, regeneration, mechanism of action, safety, efficacy, potency analysis

## Introduction

Mesenchymal stromal/stem cell (MSC) therapies have been employed in more than 800 registered clinical studies across the globe (1) and there are now >55,000 publications readily available on MSCs (2). Their profound immunomodulatory and regenerative properties have made MSCs one of the most promising and intensely pursued cellular therapies (3). Although meta-analysis of clinical trials with first-generation MSC products has demonstrated safety (4), their clinical efficacy and understanding of the underlying mechanism of action (MoA) still needs to be improved [(1, 5–10); Caplan et al.]. A better understanding of the role of patient parameters and adjunct treatment protocols is key to yield an optimal short- and long-term therapeutic benefit. Indeed, different MSC products, as well as their dosing and delivery, may be tailored for specific clinical indications according to their individual needs (6, 8, 11). To optimize next-generation MSC therapies, efforts are now underway to improve product design and delivery to patients, safety and potency assessment pre- and post-treatment, and the understanding of the exact MoA. These important topics are covered within this article collection and in the following sections we will briefly put into context the 20 articles published within this Frontiers Research Topic: “Safety, Efficacy, and Mechanisms of Action of MSC Therapies”.

## Diversification in MSC Products and Delivery

A great diversification in MSC products, treatment indications, and delivery methods has occurred over the past decade, raising many regulatory questions, and potentially entailing reevaluation of safety and efficacy for new products/applications [(1, 12); Caplan et al.]. Adjustments in manufacturing are manifold, e.g., cell expansion conditions, culture media composition, or cell priming (10). A key issue is the tissue source the MSCs are derived from, with clinical trials in the past 5 years utilizing MSCs from bone marrow (BM), adipose tissue (AT), and perinatal tissue (PT) at almost equal frequency (1).

Wilson et al. give a great overview on all aspects of MSC heterogeneity, from donor to tissue source, the role of cell isolation and *in vitro* expansion, and the regulatory considerations related to heterogeneous cell therapy. In line, Ankrum and coworkers, who recently reviewed the MSC manufacturing process for therapy (10), newly define isolation and culture conditions to better prepare MSCs for the challenging *in vivo* environments they encounter post transplantation in their title “Nature vs. Nurture” (Boland et al.).

In their review, Khan and Newsome provide an exemplary assessment on how the production process can shape the phenotype and functional properties of BM-derived multipotent adult progenitor cells (MAPC®, Athersys Inc, Cleveland Ohio) compared to various conventional BM-MSC products. Andrzejewska et al. employed multi-parameter analysis to decipher the relative impact of *in vitro* culture aging (early vs. later passage) vs. *in vivo* donor aging (adult vs. elderly donors and typically associated mild comorbidities) on BM-MSC properties in biobanking approaches.

Caplan et al. summarized how delivery methods shape the outcome of MSC therapy, differentiating between specific types of local and systemic delivery, and they further elaborate on the role of innate and adaptive immune responses, in particular cell product hemocompatibility aspects, on steering the clinical outcome. Along with earlier studies, the authors emphasize the need for prior hemocompatibility testing of cell products, if they are intended to be applied by systemic intravascular delivery [(1); Caplan et al.]. Today it is well-recognized that intravascular delivered MSCs get largely trapped in the microvascular network of the lungs and tissues. Recently developed technology to *ex vivo* perfuse transplant organs on machine perfusion allows directly delivery of MSC via arterial access. To this end, Sierra Parraga et al. report on the effects of machine perfusion conditions on the survival and functionality of MSCs.

## Safety and Efficacy of MSC

Grégoire et al. compared different MSC products derived from the three most commonly employed tissue sources (AT-, BM-, and PT-derived) in a mouse model of acute graft-vs.-host disease (GvHD). Sadeghi et al. present their results on the preclinical toxicity evaluation of clinical grade placenta-derived decidual stromal cells (DSCs) in different preclinical models. Masgutov et al. report their promising preclinical findings on peripheral nerve regeneration upon local delivery of AT-MSCs in fibrin glue. A whole different concept is to target endogenous MSC to induce immunomodulatory and regenerative effects. Ross et al. explored this concept with an anti-inflammatory extremely-low frequency pulsed electromagnetic field (PEMF) to reduce chronic inflammation for treatment of rheumatoid arthritis.

Soria-Juan et al. give a hands-on overview on their many years of experience in treatment of critical limb ischemia and diabetes with cell products, in particular AT-derived MSCs, and their optimal delivery. Avivar-Valderas et al. share their valuable data on allo-sensitization after local administration of allogeneic AT-MSCs (Darvadstrocel formerly Cx601, from Takeda/TiGenix) along with detailed mechanistic side-studies on protection and susceptibility to attack by the complement system.

## Mechanism of Action (MoA): Multifactorial Crosstalk

MSC's regenerative properties and modulation of the immune system have driven their therapeutic application for a variety of conditions. Importantly, these effects are not mediated by a single MoA; Rather, MSCs modulate different tissue and immune cells through numerous soluble immunomodulatory and trophic factors, different types of subcellular vesicles, and efferocytosis mechanisms (Ferreira et al.; Carreras-Planella et al.; Podestà et al.; Weiss and Dahlke; Weiss et al.). While being mostly studied in isolation, a better understanding on the interaction of these MoA in experimental and *in vivo* contexts remains lacking. In addition, clarification on the role of host immune cells responding to MSCs is needed, to enable the better identification of patients likely to respond to MSC-based therapies (8).

### Directionality: Direct Signaling vs. Secondary Crosstalk

A large portion of MSC's therapeutic activity is attributed to direct primary signaling through their secretome, comprising a multitude of cytokines, chemokines, growth factors, and subcellular vesicles. Ferreira et al. give a grand overview on the current knowledge of MSC's secreted mediators and how inflammatory priming influences their release. In line with this, Diedrichs et al. present their results on the clinical development of cardiac-derived MSC products and in particular the impact of interferon-gamma (IFN-g) inflammatory licensing on cell product properties in the context of allogeneic cell therapy. Another elegant study by Carreras-Planella et al. demonstrated in mechanistic fashion that the immunomodulatory effect of MSCs on B-cells is largely independent on extracellular vesicles.

Multiple experts also agree that the MoA of MSCs depends on the secondary crosstalk of therapeutic MSCs with the host tissues and in particular the host recipient immune system [(5, 6, 13, 14); Caplan et al.; Podestà et al.; Weiss and Dahlke; Weiss et al.; Yuan et al.]. Clinical effects may result from a bi-directional crosstalk between MSCs and host cells (as long as MSCs are present), and from the initiation of secondary responses of varying duration, which complicates attempts to model kinetics and dosing in “cell pharmacology” (11). In their review article, Podestà et al. decipher the impact of potential MoAs in their safety and feasibility assessment of MSC therapy for solid organ transplantation, with the aim to promote tolerance to the transplant.

### Necrobiology: Living, Apoptotic, and Dead Therapeutic Cells

Several contributed reviews elucidate how the metabolism of living cells and the physiology of apoptotic and dead cells, and thus their necrobiology, may contribute to the MoA of MSC therapeutics *in vivo* (Podestà et al.; Weiss and Dahlke; Weiss et al.;  Yuan et al.). Weiss and Dahlke delineate that direct signaling through MSC secreted factors is only part of the equation and elaborate on the role of T cells and monocytes in steering the response to viable and non-viable MSCs. A second review by Weiss et al. further elaborates how the host response to dead or dying cells and subcellular particles, and the concomitant processes of autophagy, apoptosis, mitochondrial transfer, and release of subcellular particles, may affect the therapeutic efficacy and choice of cellular therapeutics. In addition, Yuan et al. give interesting new input on the role of cell metabolism as the missing link between MSC manufacturing and therapy.

### Cryobiology: Fresh vs. Freeze-Thawed Therapeutic Cells

Regarding cell-host immune interaction, it may also be crucial to differentiate between using fresh from culture-derived metabolically active cells, as compared to freeze-thawed cells readily derived from cryostorage, which may show a transient but reversible impairment of their metabolism and cellular integrity directly after thawing [(15–19); Sierra Parraga et al.; Yuan et al.].

This is exemplified by a contribution from Sierra Parraga et al. who found altered activity of freeze-thawed compared to fresh MSCs in a model of normo-thermic machine perfusion to support transplant kidneys. Oja et al. shared their hands-on experience on how freezing steps in MSC manufacturing impact quality and cell functionality attributes, and how a short-term 24-h culture recovery post thawing can restore the full functionality of the cells. In the past years, comparisons on the effect of fresh vs. freeze-thawed cellular therapeutics have gained greater interest in the cell therapy field as a whole, since this does not only seem to be of interest/relevance for MSC therapeutics, but also for other rapidly expanding fields such as bioengineered chimeric-antigen-receptor (CAR) T-cell therapies (19, 20).

### Modulating Cell-Host Interaction by Steering Therapeutic Cell Formulation

Not only the general mode of manufacturing, but also the final steps of clinical cell formulation/delivery (e.g., cell harvesting, freezing/recovery post cryobanking, or product formulation and mode of application) could be very decisive for therapeutic safety and efficacy outcome in clinical trials (1, 8, 10, 19).

Early preclinical and clinical studies paid rather little attention to these aspects and information from publications is still scarce today. Fortunately, these aspects were discussed in great detail in the studies by Oja et al. and Sierra Parraga et al. Our own experience from early-stage trials indicated that freeze-thawed cells appear to be more prone to activate innate immune cascade systems thus being subject to faster clearance (21), which may influence their *in vivo* persistence (19). Furthermore, certain cell formulations (e.g., MSCs with low-dose heparin and human albumin instead of human blood type AB plasma) appear to give better clinical responses (22–24).

Thus, the composition of the final cell suspension including prior thawing and washing procedures, may be a key component for positively influencing cellular “pharmacodynamics” *in vivo* and should be studied with greater attention in order to optimize cellular therapeutics (1, 19, 20).

## Author Contributions

All authors listed have made a substantial, direct and intellectual contribution to the work, and approved it for publication.

### Conflict of Interest

The authors declare that the research was conducted in the absence of any commercial or financial relationships that could be construed as a potential conflict of interest.
